# A P25/(NH_4_)_x_WO_3_ hybrid photocatalyst with broad spectrum photocatalytic properties under UV, visible, and near-infrared irradiation

**DOI:** 10.1038/srep45715

**Published:** 2017-04-03

**Authors:** Linfen Yang, Bin Liu, Tongyao Liu, Xinlong Ma, Hao Li, Shu Yin, Tsugio Sato, Yuhua Wang

**Affiliations:** 1 Department of Materials Science, School of Physical Science and Technology, Lanzhou University, Lanzhou, 730000, China; 2 Institute of Multidisciplinary Research for Advanced Materials, Tohoku University, 2-1-1 Katahira, Aoba-ku, Sendai, Japan

## Abstract

In this study, a series of hybrid nanostructured photocatalysts P25/(NH_4_)_x_WO_3_ nanocomposites with the average crystallite size of P25 and (NH_4_)_x_WO_3_ of the sample was calculated to be about 30 nm and 130 nm, were successfully synthesized via a simple one-step hydrothermal method. The as-obtained samples was characterized by transmission electron microscopy (TEM), which implies that the P25/(NH_4_)_x_WO_3_ nanocomposites are fabricated with favourable nanosizd interfacial. The XPS results confirmed that the obtained sample consists of mixed chemical valences of W^5+^ and W^6+^, the low-valance W^5+^ sites could be the origin of NIR absorption. As revealed by optical absorption results, P25/(NH_4_)_x_WO_3_ nanocomposites possess high optical absorption in the whole solar spectrum of 200–2500 nm. Benefiting from this unique photo-absorption property and the synergistic effect of P25 and (NH_4_)_x_WO_3_, broad spectrum response photocatalytic activities covering UV, visible and near infrared regions on degradation of Rhodamine B have been realized by P25/(NH_4_)_x_WO_3_ nanocomposites. Meanwhile, the stability of photocatalysts was examined by the XRD and XPS of the photocatalysts after the reaction. The results show that P25/(NH_4_)_x_WO_3_ photocatalysts has a brilliant application prospect in the energy utilization to solve deteriorating environmental issues.

Today, energy crisis and environment pollution are the grim challenge of human existence. As a free, inexhaustible and sustainable energy source, solar energy has long been considered one of the most promising renewable energy sources in the world, in order to solve those problems^1^,^2^,^3^,^4^,^5^,^6^. In view of solar energy utilization, the search for semiconductor photocatalysts that can harvest the wide spectrum of solar light, from ultraviolet (UV) to near-infrared (NIR) wavelength, and achieve efficient solar energy conversion remains one of the most challenging missions^7^,^8^. Titanium dioxide (TiO_2_), a key semiconductor with tunable crystal structure (rutile, anatase, brookite), and effective photocatalytic activity^9^,^10^. The band gaps of these three TiO_2_ phases are 3.0, 3.2, and 3.25 eV, respectively. Among its useful attributes, TiO_2_ is: (i) insoluble in aqueous media, (ii) chemically and biologically inert, (iii) photostable, (iv) nontoxic, (v) inexpensive, and (vi) readily available^11^,^12^,^13^,^14^,^15^. Commercial TiO_2_ P25 is anatase/rutile nanocomposite (phase composition in ratio of 80/20 for anatase/rutile), as a result of its superior activity in the wide variety of applications^16^,^17^,^18^. However, due to a wide band gap, TiO_2_ responds primarily to UV light which only accounts for less than 5% of total solar radiation. Although the visible (Vis) light and NIR resources are abundant, under Vis light and NIR irradiation, the catalytic activity of P25 is limited by low electron transfer and high electron/hole pair recombination rates^19^. And removal of organic pollutants from wastewater through photocatalytic degradation process also suffers drawbacks of insufficient utilization on solar energy. As far as light-harvesting is concerned, most of efforts have been focused on extending the photo-responsive region of photocatalysts to match the utmost of solar energy. The landmark cases, such as N-doped TiO_2_ photocatalysts^20^, plasmonic photocatalysts^21^,^22^, morphology modification^23^,^24^,^25^,^26^,^27^, composite photocatalysts^28^,^29^,^30^,^31^, TiO_2_ films^32^, were developed to make the photocatalysts to be effective under Vis light.

What is a pity, despite all these advances, the NIR light remains seldom be utilized. To the best of our knowledge, only several kinds of nanomaterials, including up-conversion photocatalysts, Bi_2_WO_6_/TiO_2,_ Cu_2_(OH)PO_4_, Cs_x_WO_3_ and M_x_WO_3_/ZnO have tentatively been employed as NIR-driven photocatalysts until now^33^,^34^,^35^,^36^,^37^,^38^, and the aim to discover a full-spectrum-responsive photocatalyst is far from being realized. We take the up-conversion composited photocatalysts for example, it can convert NIR light to Vis light or UV light and then transfer energy to UV or Vis light active photocatalysts to induce a photocatalytic effect. However, the quantum efficiency of composited up-conversion photocatalysts is much low and the excitation source is limited to 980 nm. Moreover, most promising photocatalysts only possess UV, UV-Vis, Vis-NIR, or NIR photocatalytic activity, separately. They are not compatible to utilize full-spectrum of the solar light. Therefore, it is very significant to realized full-spectrum-responsive photocatalytic activities^39^,^40^,^41^,^42^,^43^,^44^.

For realizing the aimed full-spectrum-responsive photocatalytic properties, a broadband absorptive ability is a prerequisite for the photocatalysts. Previous research confirmed that the hexagonal tungsten bronze type compound M_x_WO_3_, which is WO_3_ doped with monovalent ions such as K^+^, Na^+^, NH_4_^+^ and others and consisting of mixed chemical valence tungsten ions (W^6+^ and W^5+^) exhibited excellent NIR absorption properties when dispersed as nanosized particles or in one-dimensional form^45^,^46^. As a novel near-infrared (NIR) shielding material, the tungsten bronze (M_x_WO_3_) have been widespread applied in smart windows, gas sensors, electrochromic materials, photocatalysts, military weapon. cancer therapy and air pollution decontamination^46^,^47^,^48^,^49^. Early on, we have exploited the synthesis of homogeneous tungsten bronze type nanocrystals of (NH_4_)_x_WO_3_, which consists of mixed chemical valence tungsten ions of W^6+^ and W^5+^. More importantly, the tungsten bronze type nanocrystals of (NH_4_)_x_WO_3_ exhibit strong optical absorption in a wide range of 200~2500 nm, covering the waveband of UV, Vis light and the whole NIR region, the low-valance W^5+^ sites are the origin of NIR absorption. However, only have been studied on optical properties of the thin film consisted of (NH_4_)_x_WO_3_ nanoparticles, and the photocatalystic properties of (NH_4_)_x_WO_3_ have not been studied^50^. As expected, in this work, we combined with efficient light absorption of ammonium tungsten bronze and P25 high catalytic activity for their nanocomposites material to achieve this goal, and we present the feasibility of realizing the advanced full-spectrum-responsive photocatalytic activity by P25/(NH_4_)_x_WO_3_ nanocomposites, which has never been studied as photocatalysts before this work.

## Results and Discussion

**Figure 1:**
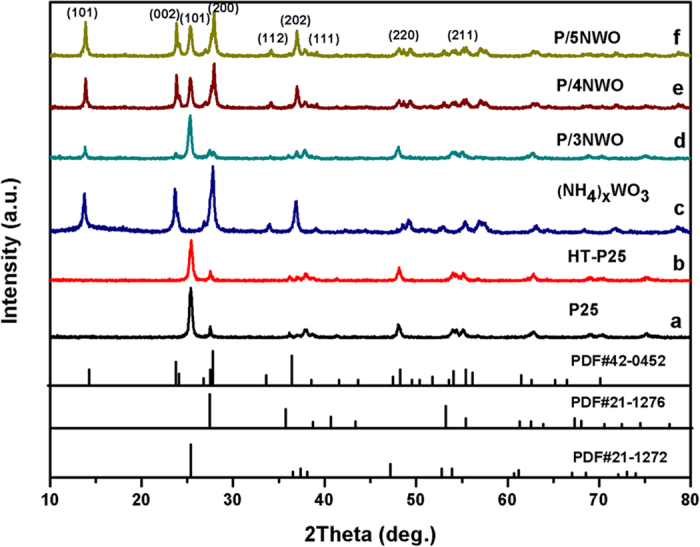
XRD patterns of pure (**a**) P25, (**b**) HT-P25, (**c**) (NH_4_)xWO_3_ and different P/NWO nanocomposites: (**d**) P/3NWO, (**e**) P/4NWO, (**f**) P/5NWO.

As shown in Fig. 1, XRD analysis has been employed for analyzing the crystalline phase of samples. The reflection in Fig. 1a matches best with the single anatase TiO_2_ (JCPDS 21-1272) and rutile TiO_2_ (JCPDS No. 21-1276) phases. As to HT-P25, its phase structure did not change after hydrothermal method (Fig. 1b). While the main peaks at 2θ values of 13.83°, 23.701° and 27.897° can be indexed respectively to (100), (002) and (200) crystal planes, which are readily indexed to the pure ammonium tungsten bronze ((NH_4_)_0.33_WO_3_; JCPDS No. 42-0452), as displayed in Fig. 1c. Furthermore, with the increasing content of (NH_4_)_x_WO_3_, the intensities of the (NH_4_)_x_WO_3_ peaks are increased obviously (Fig. 1d–f), revealing that P/NWO nanocomposites are obtained successfully during the hydrothermal process. Meanwhile, peaks related to other phases are not observed in the synthesized samples, indicating that the P25 have not reacted with the (NH_4_)_x_WO_3_.

In addition, the intensity of these XRD peaks was relative weak and half-peak breadth was wide, being consistent with the general features of the nanoparticles. Enlighten by this, the average crystallite size (D) of P25 and (NH_4_)_x_WO_3_ of the sample was calculated to be about 30 nm and 130 nm, using the well-known Scherrer equation:


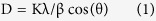


where λ is the wavelength of the X-ray radiation (λ = 0.15418 nm), Κ is the Scherrer constant (Κ = 0.89), θ is the Bragg angle of the X-ray diffraction peak, and β is the line broadening at half the maximum intensity (FWHM, in radians) of the (101) plane of P25 and the (200) plane of (NH_4_)_x_WO_3_ of sample.

**Figure 2:**
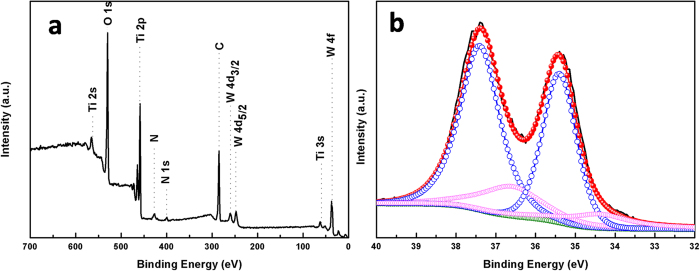
XPS spectra of P25/(NH_4_)_x_WO_3_ nanocomposites (**a**), full range XPS spectra (**b**) deconvolution of W 4 f core-level spectrum with peaks corresponding to W^6+^, W^5+^ oxidation states.

The chemical composition and valence state of the P/NWO nanocomposites were examined by X-ray photoelectron spectroscopy (XPS). The fully scanned spectra clearly reveal that elements of Ti, N, W, O and C existed in the sample (Fig. 2a). The presence of carbon element in the final product could be related to the residual chemically or physically adsorbed organics originating from the solvent molecules. For tungsten, a complex energy distribution of W4f photoelectrons was obtained as shown in Fig. 2b. The obtained XPS curve could be fitted into two spin-orbit doublets, corresponding to two different oxidation states of W atoms. The main peaks, having a W 4f_5/2_ at 37.5 eV and a W 4f_7/2_ at 35.4 eV, could be attributed to the W atoms being in a + 6 oxidation state. The second doublet, with a lower binding energy at 34.4 eV and 36.5 eV, could be ascribed to the emission of W 4f_5/2_ and W 4f_7/2_ core levels from the W atoms in an oxidation state of + 5. These results on the core level of tungsten ions in tungsten bronze are in good agreement with reported values^51^.

**Figure 3:**
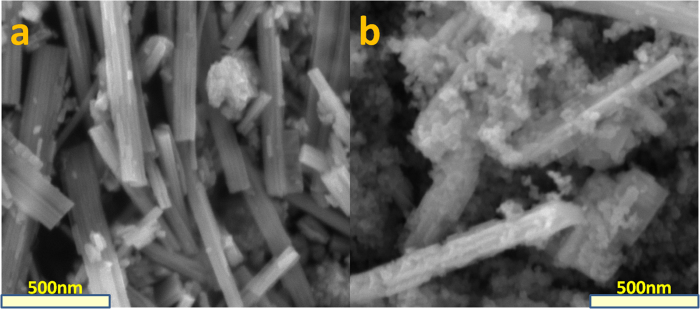
SEM image of (**a**) (NH_4_)_x_WO_3_ (**b**) P/4NWO.

The morphology of the as-prepared samples was characterized by SEM, which is shown in Fig. 3. As shown in Fig. 3a, the (NH_4_)_x_WO_3_ exhibit smooth surface, numerous nanorods with the diameter of the nanorods ranged from 100 to 200 nm. Figure 3b shows the SEM image of the P/4NWO nanocomposites, and it could be clearly seen that TiO_2_ particles are attached to the surface of (NH_4_)_x_WO_3_ nanorods, indicating the intimate contact between P25 and (NH_4_)_x_WO_3_.

**Figure 4:**
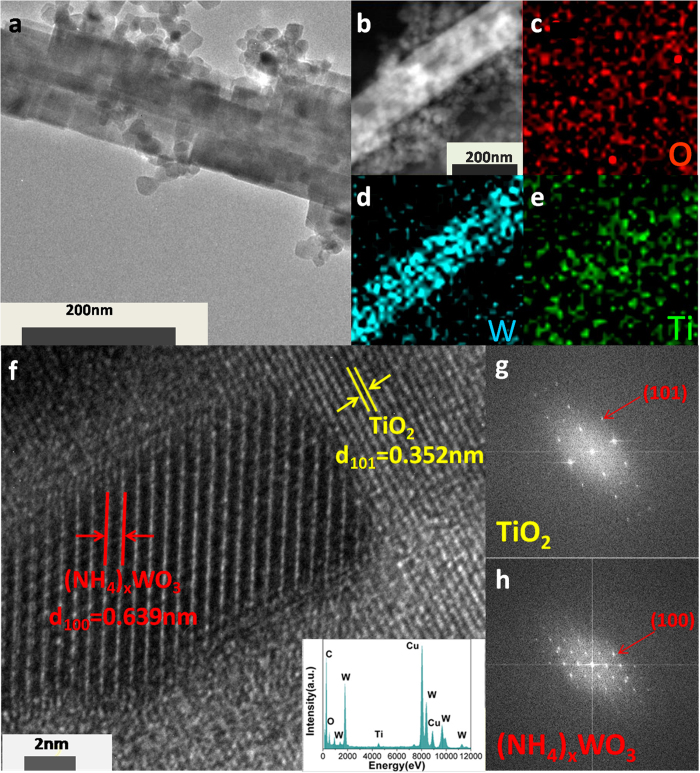
(**a**) TEM images of the obtained P/4NWO. (**b**) The STEM image of the element distribution map. (**c–e**) The representative element mapping images of nanocomposite P/4NWO with the same scale bar of 200 nm. (**f**) The HRTEM images of as-synthesized P/4NWO nanocomposite (the inset shows EDX spectrum of P/4NWO nanocomposite) and (**f,g**) corresponding FFT patterns.

To further obtain the microscopic morphology and structure information, the TEM and HRTEM analysis of as-synthesized P/4NWO nanocomposites have been performed. As shown in Fig. 4, the TEM images of P/4NWO nanocomposites (Fig. 4a) show the specific rod-like morphology with some nanoparticles attached, which is in accordance with SEM results (Fig. 3b). In addition, the elemental composition of P/4NWO has been investigated by EDX spectrum (insert Fig. 4f) and elemental mapping analysis (Fig. 4d–e). Clearly, the homogeneous distribution of Ti, O, and W elements suggests the co-existence of (NH_4_)_x_WO_3_ and TiO_2_. Moreover, to further confirm the co-presence of the TiO_2_ and (NH_4_)_x_WO_3_ in P/NWO nanocomposites, the HRTEM image of the magnified view is given in Fig. 4f. The distance of 0.639 nm and 0.352 nm between the adjacent lattice fringes can be assigned to the (100) plane of hexagonal (NH_4_)_x_WO_3_ and the (101) plane of anatase TiO_2_ nanocrystals, respectively. FFT patterns of the interface between TiO_2_ and (NH_4_)_x_WO_3_ were also appended in Fig. 4e and f, revealing that TiO_2_ and (NH_4_)_x_WO_3_ are of tetragonal and hexagonal crystal structures, respectively. Obviously, the P/4NWO nanocomposites are formed with favourable nanosizd interfacial contact so as to exhibit excellent photogenerated carriers transfer and separation properties of photocatalytic degradation.

The higher BET specific surface area is beneficial to enhancing the photocatalytic activity of the composites study, which were measured and are listed in Table 1. BET specific surface area of HT-P25 (50.7 m^2^/g) did not reduce via the hydrothermal treatment. The (NH_4_)_x_WO_3_ nanorods exhibited a relatively lower BET specific surface area (11.4 m^2^/g) than P25(51.2 m^2^/g). The surface area of the P/NWO nanocomposites increased to three times more than (NH_4_)_x_WO_3_ nanorods when (NH_4_)_x_WO_3_ nanorods was combined with P25. The higher adsorption capacity could lead to the easier and faster photocatalytic degradation process because the photocatalytic reaction is a surface-based process.

**Figure 5:**
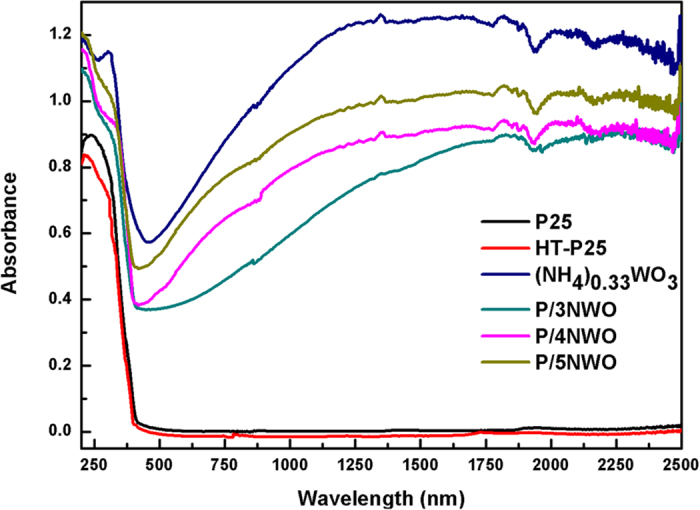
UV–Vis-NIR absorption spectra of a series of the samples.

The optical property is another critical factor on determining whether certain material is a potential candidate as photocatalyst. The UV-Vis-NIR absorption spectra of P25 and as-obtained powder were measured and shown in Fig. 5. It clearly reveals that TiO_2_ shows a strong absorption in the UV region, and the Eg of TiO_2_ is approximately 3.05 eV, which corresponds to the absorption of wavelength <400 nm. As for (NH_4_)_x_WO_3_ and P/NWO particles, the spectrum shows a broad and strong light absorption in the whole solar region of 200–2500 nm, especially for the NIR light of 800–2500 nm. This phenomenon suggests that the P/NWO nanocomposites have a potential photocatalytic activity under irradiation of UV, Vis and NIR light, which also is highly necessary for investigating the aimed full-spectrum-response photocatalytic activity.

**Figure 6:**
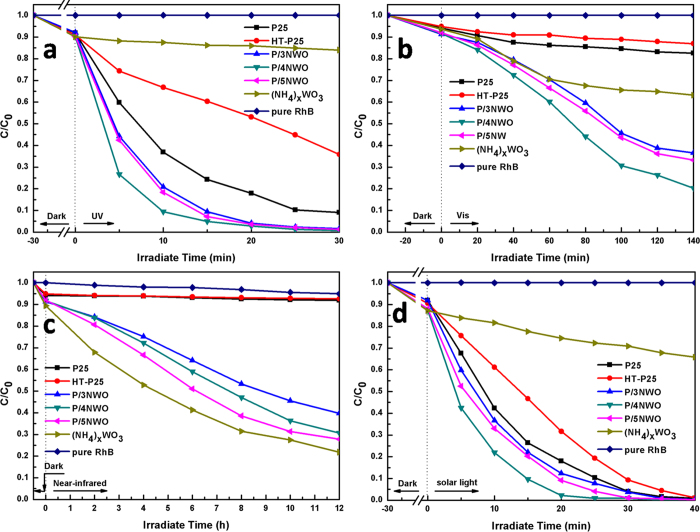
Photocatalytic degradation of RhB in the presence of P25, HT-P25. (NH_4_)_x_WO_3_ nanorods and P25/(NH_4_)_x_WO_3_ nanocomposites and pure RhB under (**a**) UV (**b**) Vis (**c**) NIR (**d**) solar light.

To prove the photocatalytic activity of the P/NWO nanocomposites, the decomposition of RhB in water under UV, visible light, near-infrared light and simulated solar light irradiation as a function of irradiation time were investigated (Fig. 6a–d). For comparison, decomposition abilities of P25, the processed P25 (HT-P25), (NH_4_)_x_WO_3_ nanorods and RhB with absence of photocatalysts were measured under the same experimental conditions.

As shown in Fig. 6, (NH_4_)_x_WO_3_ nanorods have very limited UV photocatalytic activities (Fig. 6a). When combined P25 with (NH_4_)_x_WO_3_ nanorods, the UV photocatalytic performance of the P25/NWO nanocomposites is dramatically improved, especially when the value of y is 40%. The corresponding decomposition rate increases to nearly 100% after 20 min UV irradiation (Fig. 6a), which is much higher than that of P25 (83%), HT-P25 (47%), and (NH_4_)_x_WO_3_ nanorods (8%). Similar to the results in the previous works^52^, P25 exhibits poor visible photocatalytic activities, and P/NWO nanocomposites possess good visible photocatalytic degradation property. The corresponding RhB degradation degree under 140 min visible light irradiation for P25, HT-P25 and (NH_4_)_x_WO_3_ nanorods is 17.5%, 13.3% and 37.8% respectively(Fig. 6b). Surprisingly, the decomposition rate of the P25/4NWO nanocomposites increases to 81% after visible light irradiation for 140 min.

Here, (NH_4_)_x_WO_3_ nanorods exhibited good near-infrared catalytic activity. The RhB degradation degree for (NH_4_)_x_WO_3_ nanorods under 12 h near-infrared light irradiation is 68%. It is significant that P/NWO nanocomposites possess enhanced near-infrared photocatalytic activities compared with P25 and HT-P25, and the RhB degradation degree for P25 and HT-P25 under 12 h near-infrared light irradiation is 60% (P/5NWO) (Fig. 6c). In contrast, P25 and HT-P25 nearly have no near-infrared degradation ability to RhB. The corresponding RhB degradation rate for P25 and the processed P25 is only 2.1% and 1.8% in the same conditions. We also checked the photocatalytic property of the above photocatalysts under conditions similar to natural solar light irradiation. The natural solar light was generated by a solar-simulator (300 W xenon arc lamp with AM 1.5 G filter, 100 mW cm^−2^, microsolar300), the photocatalytic degradation efficiency under solar-light follows the order P/4NWO > P/5NWO > P/3NWO > P25 > HT-P25 (Fig. 6d). Moreover, with regard to UV light photodegradation (Fig. 6a), no photolysis of RhB is observed after 30 min UV light irradiation with the absence of photocatalysts, and similar phenomena have been observed in the other light photodegradation (Fig. 6b–d). These results further confirm that the photocatalytic properties of above samples are attributed to the photocatalysis reaction, instead of the self-degradation of RhB solution. So far, the full-spectrum-responsive photocatalytic properties of P/NWO nanocomposites were demonstrated. To the best of our knowledge this phenomenon has never been reported.

Then, to investigate the stability of (NH_4_)_x_WO_3_ in the solution under tested conditions the concentration of tungsten element of the filtrated photocatalytic reaction solution was measured. As shown in Fig. S1, ICP analysis revealed that in the presence of the W elements is 0.09, 1.22, 3.05 and 1.02 mg/L in after UV, visible, near-infrared and solar light photocatalytic reaction, respectively. The results indicate the loss of (NH_4_)_x_WO_3_ is very few in photocatalytic reaction.

**Figure 7:**
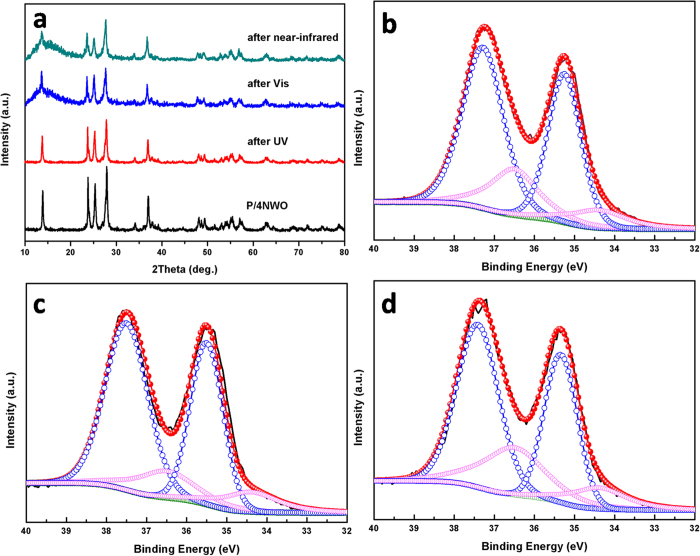
(**a**) XRD patterns of P/4NWO after the photocatalytic reactions; W4f core-level XPS spectra of P/4NWO (**b**) after the UV photocatalytic reaction (**c**) after the Vis light photocatalytic reaction (**d**) after the near-infrared photocatalytic reaction.

In addition, the XRD and XPS of (NH_4_)_x_WO_3_ after the photocatalytic reaction were examined. As shown in Fig. 7a, it could be seen that the XRD patterns of P/4NWO samples before and after the reaction were essentially identical except for decreasing of the intensities of the diffraction peaks after the photocatalytic reactions. Fig. 7b–d shows the XPS spectra of W 4 f profiles of the P/4NWO after the photocatalytic reactions. The results shown that there are still two spin-orbit doublets in this spectrum, which can be indexed to W 4f_7/2_ and W 4f_5/2_: the peaks at 34.4 and 36.5 eV are attributed to the W^5+^, while the peaks at 35.4 eV and 37.5 eV are assigned to W^6+^, which still reaches a good agreement with the reported results^51^. These results further indicate the (NH_4_)_x_WO_3_ act as a stable photocatalyst under UV, visible, near infrared and even solar light illumination.

**Figure 8:**
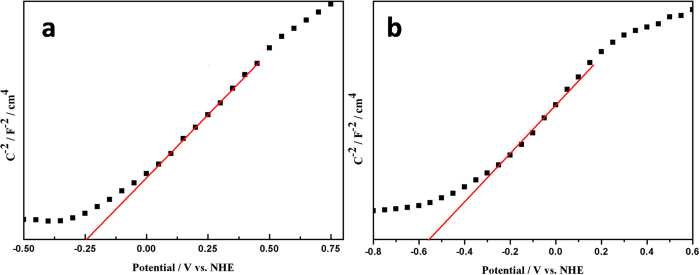
Mott-Schottky plots for (**a**) TiO_2_ and (**b**) (NH_4_)_x_WO_3_ collected at a frequency of 1000 Hz in the dark.

**Figure 9:**
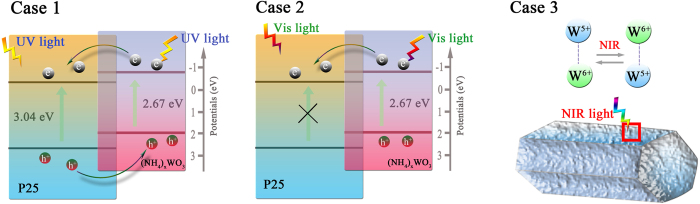
The photocatalytic mechanism of P25/(NH_4_)_x_WO_3_ nanocomposites.

Recently, photocatalytic degradation mechanism of dye under the UV or Vis light irradiation on semiconductors has been well established as an oxidative process in which three consecutive steps are involved. Firstly, light with higher energy than bandgap of semiconductor is absorbed and afterwards induces a transition of electrons from the valence band to the conduction band, leaving an equal number of holes in the valence band. Secondly, the excited electrons and holes migrate to the surface. Thirdly, the superficial photogenerated electron/hole pairs produce several reactive intermediate species for destruction of dye molecules. Generally, the photogenerated electrons are scavenged by dissolved oxygen, and then superoxide (O_2_•^−^) would be obtained first, followed by formation of other reactive oxygen species including hydroperoxyl radical (HOO•), H_2_O_2_, or hydroxy radical (OH•)^53^. All these reactive oxygen species possess sufficient energy for oxidation of pollutants. Based on the above discussion, the enhanced UV and Vis photocatalytic activities of P/NWO nanocomposite are contributed to the improvement capacity of light absorption and efficient separation of photo-induced carriers. To clarify the possible mechanism of the enhancement of photocatalytic activity, the positions of conduction band (CB) and valence band (VB) of P25 and (NH_4_)_x_WO_3_ are determined by flat-band potentials (V_fb_) and UV-Vis-NIR absorption spectra, as displayed in Fig. 8. The V_fb_ can be quantified by the Mott–Schottky equation:


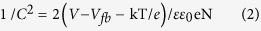


where *C* is the total measured capacitance, V is the electrode applied potential, V_fb_ is the flat band potential, *ε*_*0*_ is the vacuum permittivity, *ε* is the dielectric constant of the material, e is the electron charge, k is the Boltzmann constant, T is the temperature, and N is the acceptor concentration. According to the Mott–Schottky equation, a linear relationship of C^−2^ vs. V can be observed (Fig. 8a and b) and the intercepts of the straight lines with the potential axis indicate the V_fb_ values of P25 and (NH_4_)_x_WO_3_ are −0.24 V vs NHE and −0.55 V vs NHE^54^, thus the CB of P25 and (NH_4_)_x_WO_3_ are −0.44 V vs NHE and −0.75 V vs NHE, respectively^55^. Furthermore, combining with the bandgap energy of P25 (~3.05 eV) and (NH_4_)_x_WO_3_ (~2.67 eV) as displayed in Fig. S2a and b, the energy level diagrams of P/NWO heterojunction with the presence of TiO_2_, (NH_4_)_x_WO_3_ are obtained and displayed in Fig. 9. As shown in Fig. 9 (case 1), under UV light irradiation, both P25 and (NH_4_)_x_WO_3_ could be excited to generate electrons and holes. Electrons photoexcited from (NH_4_)_x_WO_3_ transfer to the CB of TiO_2_, and holes photoexcited from P25 transfer from the VB of P25 to that of (NH_4_)_x_WO_3_. This transfer process is thermodynamically advantageous because both the CB and VB of (NH_4_)_x_WO_3_ are more negative than those of P25. Under the circumstances, more effective transfer and separation of the photoinduced carriers between P25 and (NH_4_)_x_WO_3_ interfaces, resulting in markedly improved UV photocatalytic activity. When the P/NWO nanocomposite is irradiated by Vis light, only (NH_4_)_x_WO_3_ could act as a sensitizer benefits by its narrow band gap. As displayed in Fig. 9 (case 2), under Vis light illumination, electrons in the VB of (NH_4_)_x_WO_3_ could be excited to a higher CB, and then transfer to the CB of P25. Meanwhile, the photogenerated holes accumulated in the VB of (NH_4_)_x_WO_3_ will accelerate the decomposition of organic pollutants into non-toxic substance. In such a way, the photoinduced electrons and holes could be separated effectively, so as to settle the high recombination probability of the photo-induced carriers in (NH_4_)_x_WO_3_ and greatly enhance photocatalytic activity under Vis light irradiation.

As for NIR light, it could be clearly seen from Fig. 9 (case3) that the NIR-driven photocatalytic activity of P/NWO nanocomposites and (NH_4_)_x_WO_3_ nanorods are only attributed to the low-valance W^5+^ sites of (NH_4_)_x_WO_3_ nanorods. When P/NWO nanocomposites were irradiated by the NIR, W^5+^ sites as photosensitive sites can induce one electron out from low-valence W^5+^ site and then this W^5+^ will convert to W^6+^ (Eq. (3)). In our previous work, we have confirmed the probability that the NIR generated electron from W^5+^ site can also transfer to third party in the photoreaction system^37^. Subsequently, photogenerated electrons could be trapped by absorbed O_2_ to form O_2_^-^, followed by the generation of •OH, •OOH and ^1^O_2_ (Equations (4) and (5))^56^. On the other side, the W^6+^ sites can react with OH^−^ and return to W^5+^, realizing a full photocatalytic circle. Finally, the reactive species, including •OH, •OOH and ^1^O_2_ all possess sufficient energy for oxidation of dyes (Equation (7)).


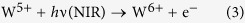



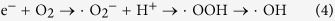



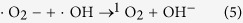



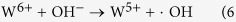



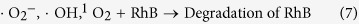


In conclusion, as exactly in accordance with the essence of Eq. (3), when the NIR light irradiates on (NH_4_)_x_WO_3_ nanorods, induces one electron to escape from its W^5+^ site and then oxidizes the original W^5+^ into W^6+^. If only (NH_4_)_x_WO_3_ presents, the only way where the neighbouring W^6+^ will be reduced into a new W^5+^ site by this electron, accompanying with generation of a phonon. However, in our case of photocatalytic reaction in the aqueous dye solution, another way for the NIR induced free electrons is scavenged by dissolved oxygen to produce various reactive species. Therefore, the proposed photocatalytic mechanism (as shown in Equations (3)–(7)) gives a sound explanation on NIR-driven photocatalytic activity of P/NWO nanocomposites and (NH_4_)_x_WO_3_ nanorods.

In summary, full-spectrum-responsive photocatalytic activities have been realized by a stable P/NWO hybrid photocatalysts, which were successfully synthesized via a simple one-step hydrothermal method, and P/NWO nanocomposites are an excellent optical absorber that can prominently harvest the light in a wide range of 200–2500 nm. The high UV and visible light photodegradation activity of P/NWO nanocomposites can attribute to the synergy of P25 and (NH_4_)_x_WO_3_ nanorods, such as large extended absorption of solar light, high electron–hole separation efficiency and stronger adsorptivity of pollutants. The low-valance W^5+^ sites are the origin of NIR absorption, also upon which the free electrons could be generated under the NIR irradiation and subsequently formed reactive oxygen species for photodegradation of RhB molecules. This work realizes utmost match of solar energy for the aimed photocatalytic reaction and this result is of significance in the utilization of all solar band energy for efficiently removal of organic dyes, whether UV, visible or near infrared light.

## Methods

### Preparation of P25/(NH_4_)_x_WO_3_ nanocomposites

All reagents were of analytical grade and used without further retreatment. First of all, different amounts of the ammonium paratungstate hydrate were dissolved into 40 ml ethylene glycol (EG) at about 190 °C. After cooling down this EG solution to the room temperature, 0.4 g of P25 were added into mixed solution, stirring and ultrasonic dispersing for a certain amount of time. Next, 20 ml acetic acid was added into the solution and stirred 30 min. Then, the obtained solution was transferred into a Teflon-lined autoclave of 100 ml internal volume, followed by hydrothermal reaction in an electric oven at 200 °C for 40 h. After the reaction, the powder was centrifuged, washed 4 times with deionized water and ethanol, respectively, and finally dried at 60 °C. As-obtained powder are P25/(NH_4_)_x_WO_3_(y%), with y = 30,40,50, and it represents the weight of the (NH_4_)_x_WO_3_ in the nanocomposites. These samples are labelled as P(P25)/3NWO[(NH_4_)_x_WO_3_], P/4NWO, and P/5NWO, respectively.

As a comparison, control samples were prepared in the absence of the ammonium paratungstate (HT-P25) or P25 ((NH_4_)_x_WO_3_) according to the above procedure.

### Characterization

The phase purity of samples was analyzed by X-ray powder diffraction (XRD) using a Bruker D2 PHASER X-ray diffractometer with graphite monochromator using Cu Kα radiation (λ = 1.54184 Å) at room temperature. X-ray photoelectron spectroscopy (XPS, PHI-5702, Physical Electronics) was performed using a monochromated Al Ka irradiation. The chamber pressure was ~3 × 10^−8^ Torr under testing conditions. The morphologies of different samples were observed by transmission electron microscopy (TEM) and high-resolution transmission electron microscopy (HRTEM, FEI Tecnai F30, operated at 300 kV). Diffuse reflectance UV-Vis-NIR absorption spectra were measured using a Perkin Elmer Lambda 950 spectrometer, while BaSO_4_ was used as a reference. The specific surface area of the samples was measured by the dynamic Brunauer–Emmett–Teller (BET) method, in which N_2_ was adsorbed at 180 °C using a Micromeritics ASAP 2000 system. The concentration of tungsten element of the solution after the photocatalytic reaction were determined by inductively coupled plasma (ICP) elemental analyses from Optial Emission Spectrometer, Varian 725-ES.

### Evaluation of photocatalytic activity

The photocatalytic activity of the sample was evaluated by measuring the degradation ratio of Rhodamine B (RhB). The initial concentration of RhB solution was 20 mg/L and 10 mg/L, the amount of photocatalysts was 0.05 g and 0.10 g per 50 mL of RhB solution, respectively. After the sample suspension was stirred for 30 min in the dark to realize the adsorption equilibrium. Respectively, the photocatalytic reaction was started using a 500 W mercury lamp as UV light source, use a 350 W xenon lamp as Vis light source for the same experiment, a 300 W infrared lamp as the nearinfrared light source where the λ < 800 nm were filtered out during nearinfrared light photocatalysis, and a solar-simulator (300 W xenon arc lamp with AM 1.5 G filter, 100 mW cm^−2^, microsolar300) used as the solar light source. In addition, the degradation of RhB with absence of photocatalysts was measured under irradiation of different spectral range. A series of a certain volume of suspension were withdrawn at selected times for analysis. After recovering the catalyst by centrifugation, the concentration of RhB solution was analyzed by measuring the light absorption of the clear solution at 554 nm using a spectrophotometer (Perkin Elmer Lambda 950).

### Photoelectrochemical Measurements

The flat-band potentials (Vfb) were determined from Mott–Schottky plots by an electrochemical analyzer (CS 310, Wuhan Corrtest Instrument Co. Ltd.) in a standard two-electrode system using the pure P25, and (NH_4_)_x_WO_3_ (effective area was 1 cm^2^) as working electrodes, a Pt foil and an Ag/AgCl (saturated KCl) electrode were used as the counter electrode and reference electrode, respectively. The photoanode was suspended into Na_2_SO_4_ (0.1 mol L^−1^) aqueous solution. The Mott–Schottky measurements were performed at a fixed frequency of 1000 Hz with 10 mV amplitude, at various applied potentials.

## Additional Information

**How to cite this article:** Yang, L. *et al*. A P25/(NH_4_)_x_WO_3_ hybrid photocatalyst with broad spectrum photocatalytic properties under UV, visible, and near-infrared irradiation. *Sci. Rep.* **7**, 45715; doi: 10.1038/srep45715 (2017).

**Publisher's note:** Springer Nature remains neutral with regard to jurisdictional claims in published maps and institutional affiliations.

## Supplementary Material

Supplementary Information

## Figures and Tables

**Table 1:** Specific surface area (S_BET_) of the prepared samples and P25 sample.

Sample	S_BET_(m^2^/g)
P25	51.2
HT-P25	50.7
(NH_4_)_x_WO_3_	11.4
P/3NWO	47.6
P/4NWO	44.1
P/5NWO	40.9
