# Supplementary material for: A P25/(NH4)xWO3 hybrid photocatalyst with broad spectrum photocatalytic properties under UV, visible, and near-infrared irradiation
Source: Sci Rep. 2017 Apr 3;7:45715. doi: 10.1038/srep45715 (PMC5377943; doi:10.1038/srep45715)
Supplement: Supplementary Information [file srep45715-s1.doc]

**A P25/(NH_4_)_x_WO_3_ hybrid photocatalyst with broad spectrum photocatalytic properties under UV, visible, and near-infrared irradiation**

**Linfen Yang**^1^**, Bin Liu**^1^,**^*^, Tongyao Liu^1^, Xinlong Ma**^1^**, Hao Li**^1^**, Shu Yin^2^, Tsugio Sato^2^ & Yuhua Wang**^1^,**^*^**

^1^ Department of Materials Science, School of Physical Science and Technology, Lanzhou University, Lanzhou, 730000, China

^2^ Institute of Multidisciplinary Research for Advanced Materials, Tohoku University, 2-1-1 Katahira, Aoba-ku, Sendai, Japan

^*^ Corresponding author: Yuhua Wang, Ph.D, Professor; Bin Liu, Ph.D.

Tel: +86-931-8912772; Fax: +86-931-8913554

E-mail address: [wyh@lzu.edu.cn;](mailto:wyh@lzu.edu.cn;) liubin@lzu.edu.cn.

**Supplementary Information**


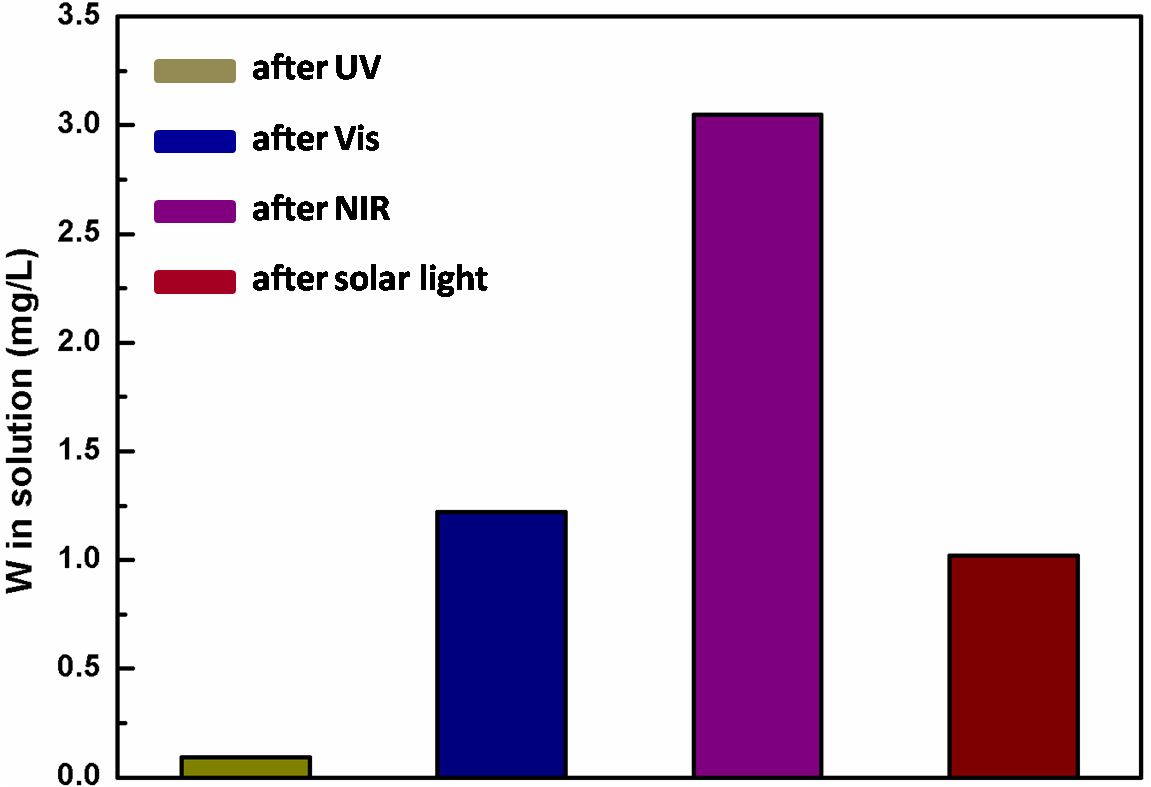


**Fig. S1.** Concentration of W element in the solution after photocatalytic reactions.


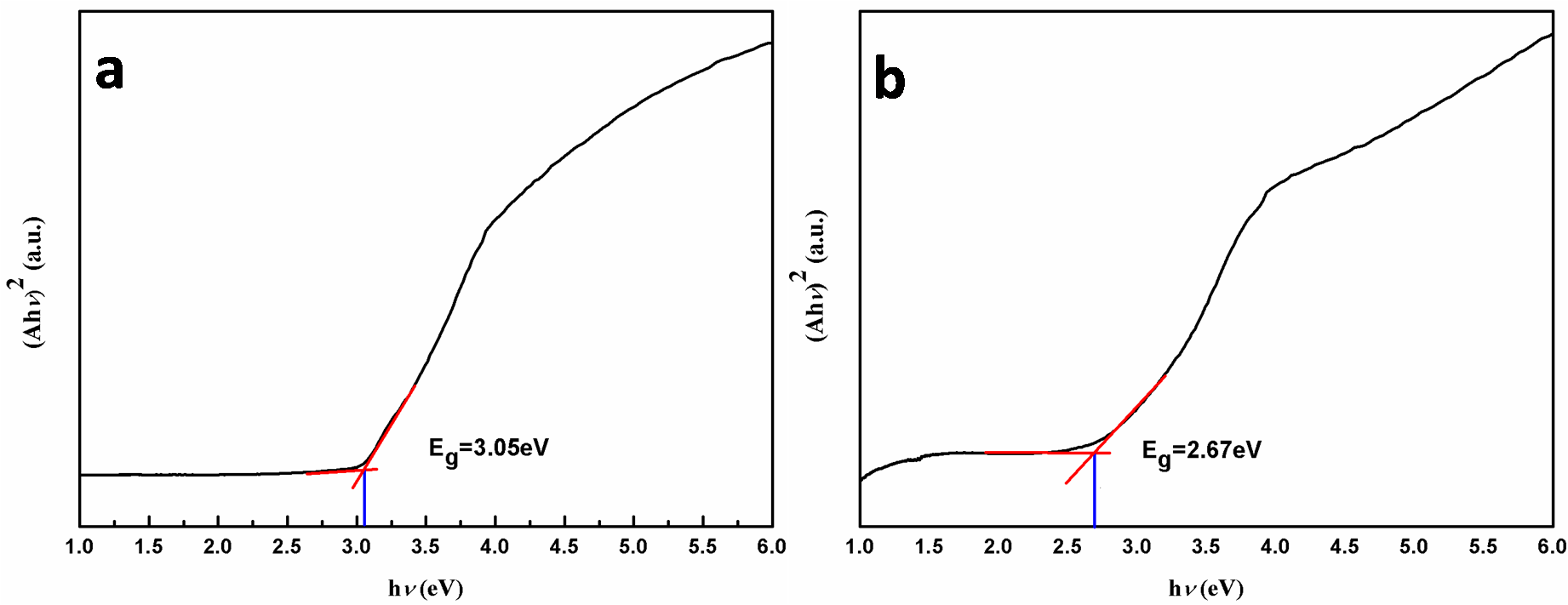


**Fig. S2.** The plot of (αhv)^2^ versus photo energy for the band gap energy of (a)TiO_2_ and (b) (NH_4_)_x_WO_3_.
